# Study of the Analgesic Activity of the Aqueous and Methanolic Extracts of Fresh Rhizome of Zingiber officinale in Wistar Rats

**DOI:** 10.7759/cureus.74219

**Published:** 2024-11-22

**Authors:** Dr. Prathamesh V Pakale, Chitra Khanwelkar, Vandana Thorat, Sujata Jadhav, Devkumar D Tiwari

**Affiliations:** 1 Department of Pharmacology, Krishna Institute of Medical Sciences, Krishna Vishwa Vidyapeeth (Deemed to be University), Karad, IND

**Keywords:** analgesic treatment, continued aspirin therapy, ginger extract, herbal pharmacology, wistar rats

## Abstract

Introduction

The study was done to evaluate the analgesic activity of the aqueous and methanolic extracts of fresh rhizome of *Zingiber officinale (Z. officinale)*. The study objectives included evaluating and comparing the analgesic activity of both extracts at two different doses with that of the standard drug (aspirin) in Wistar rats using the rat tail-flick method.

Method

The study was conducted after receiving approval from the animal ethics committee. The animals were divided into six groups of six rats each. The control group received 0.2 ml normal saline IP whereas the test groups received aspirin (150 mg/kg), aqueous extract of fresh rhizome of *Z. officinale* (100 and 200mg/kg), and methanolic extract of fresh rhizome of *Z. officinale* (100 and 200 mg/kg). The rat tail-flick method was used to investigate the analgesic activity.

Result

The present study clearly showed the significant analgesic activity of the aqueous and methanolic extracts of fresh rhizome of *Z. officinale *(p<0.05) via the rat tail-flick method compared to the control. Moreover, their activities were comparable to aspirin.

Conclusion

The aqueous and methanolic extracts of *Z. officinale* have significant analgesic activity properties.

## Introduction

Apart from their anti-inflammatory properties, non-steroidal anti-inflammatory drugs (NSAIDs) are also non-specific analgesics and can be used for acute or chronic pain [1]. However, they can cause adverse effects like peptic ulcer, nephrotoxicity, and hepatic damage, whereas ginger has digestive, antiemetic, and hepatoprotective properties [1,2].

Ginger [Botanical name: *Zingiber officinale* (*Z. officinale); *Family: Zingiberacae] is a plant distributed worldwide [2]. It has been used as a spice and a flavoring agent in food. It has also been used by traditional Indian and Chinese medicine for more than 25 centuries. It is grown widely in India, Jamaica, Mexico, and Hawaii. It is an underground root or rhizome and is used in traditional medicine for its antiemetic effect, improvement in blood circulation and digestion, etc. [3]. Various animal studies, pilot studies in humans, and clinical trials suggest the analgesic [4,5], anti-inflammatory [6,7], hepatoprotective [8], hypouricemic [9], antidiabetic [10], and anticancer [8] effects of either the crude extract or pure gingerol. The use of ginger extract for acute and chronic analgesic and anti-inflammatory purposes is not established in modern medicine and is not documented in textbooks. The many uses of the crude ginger extract are mentioned only in the traditional medicine of different countries and are not yet proven. Though some experimental studies have shown its analgesic effects in animals, few report no activity [4,5]. If the analgesic effect of ginger extract is proven, it will be a step toward the availability of a new and safe drug that will be useful for patients suffering from pain and inflammation.

## Materials and methods

Animals

Adult Wistar rats of either sex weighing 150-200 gms were procured from the Central Animal House, Krishna Institute of Medical Sciences (KIMS), Krishna Vishwa Vidyapeeth (KVV), Karad. A total of six groups of animals with six animals each were used for the experiments. A total of 36 Wistar rats were used over the six-month study period. All the experiments were performed in the Central Animal House, KIMS.

Drugs

**Figure 1:**
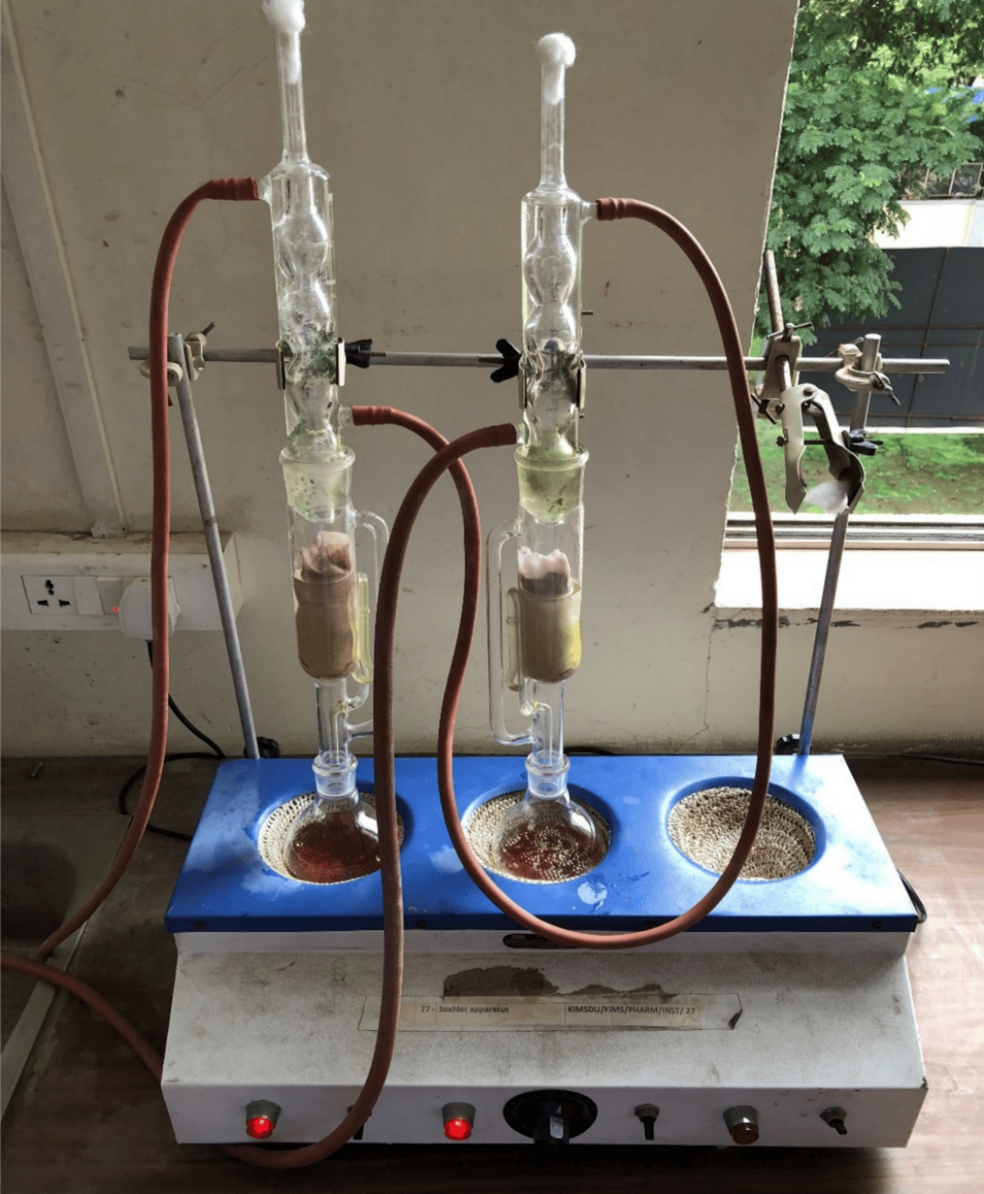
The Soxhlet apparatus used for preparing ginger extracts at the Department of Pharmacology, KIMS, KVV, Karad Manufacturer details: ISKO®, Gupta Scientific & Glass Works, Haryana, India; KIMS: Krishna Institute of Medical Sciences; KVV: Krishna Vishwa Vidyapeeth

Fresh rhizome of *Z. officinale* was obtained from the local market. It was used after authentication by a botanist. The rhizome was washed with tap water and shade-dried and then cut into pieces and put in the Soxhlet apparatus (ISKO®, Gupta Scientific & Glass Works, Haryana, India). The aqueous and methanolic extracts of both powders were prepared using the Soxhlet apparatus shown in Figure 1. All the dried extracts were dissolved in 0.9% normal saline for an intraperitoneal injection.

The extracts and the standard drug were used in two doses as mentioned in Table 1.

**Table 1:** Experimental groups and doses i.p: Intraperitoneal; mg/kg: milligram per kilogram; *Z. officinale: Zingiber officinale*

Group	Name of drug /extract	Short form used	Dose
Group I	0.9% normal saline	NS	0.2 ml i.p
Group II	Aspirin (Standard Control)	ASP	150 mg/kg i.p
Group III	Aqueous extract of fresh rhizome of *Z. officinale*	AFZ	100 mg/kg i.p
Group IV	Aqueous extract of fresh rhizome of *Z. officinale*	AFZ	200 mg/kg i.p
Group V	Methanolic extract of fresh rhizome of *Z. officinale*	MFZ	100 mg/kg
Group VI	Methanolic extract of fresh rhizome of *Z. officinale*	MFZ	200 mg/kg

The doses were selected as per previous research on the anti-inflammatory activity of the ginger extract [11]. An acute toxicity study was done and no mortality was seen up to 2000 mg/kg of methanolic extract of *Z. officinale*. All the experiments were conducted after approval from the Institutional Animal Ethics Committee of KIMS, KVV, Karad (certificate No: IAEC/KIMS/2019/09). Experiments were conducted as per the Committee for the Purpose of Control and Supervision of Experiments on Animals (CPCSEA) guidelines.

Evaluation of the analgesic activity

**Figure 2:**
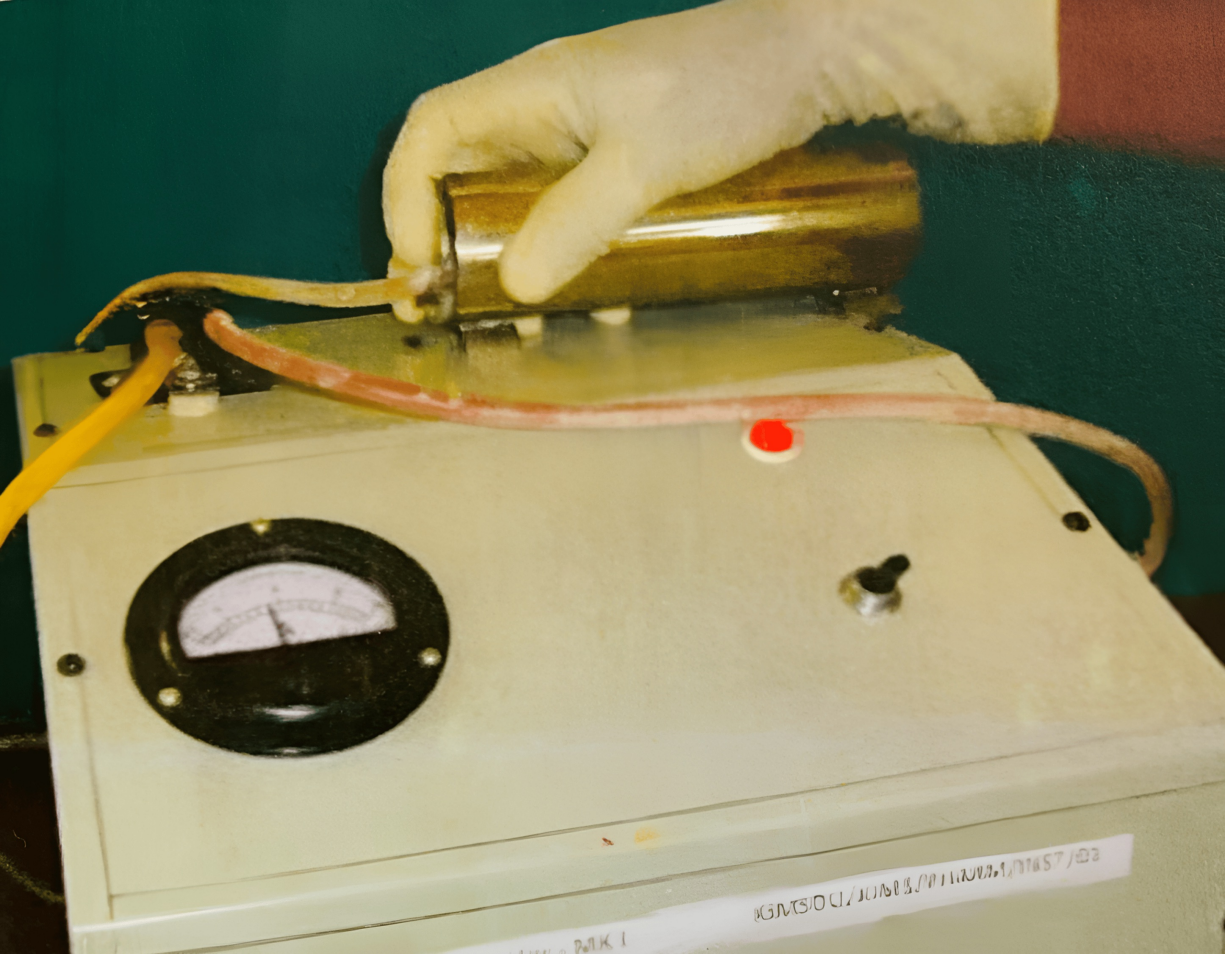
Radiant heat analgesiometer showing rat tail flick response Analgesiometer: Techno Electronics, Lucknow, India.

A radiant heat analgesiometer (Techno Electronics, Lucknow, India) was used for evaluating the analgesic activity (Figure 2). The animals were placed in a restrainer which had an aperture for the tail. A timer was started simultaneously and the time taken by the animal to withdraw (flick) its tail was taken as the endpoint of the test. A cut-off time of 20 seconds was followed to avoid tissue damage. The loss of the tail flick response by the rat was taken as the end point and a positive response. The reaction time in seconds was used as the unit for measurement of pain and an increase in reaction time was indicative of analgesia. The rats received the study drugs (saline/aspirin/*Z. officinale *extract) and were tested for the tail-flick response at zero, 30, 60, and 120 minutes after the injection [12].

Statistical analysis

Results were expressed as mean ± standard deviation (SD). Statistical analysis was performed using one-way analysis of variance (ANOVA) followed by Dunnett’s t-test for the post-hoc analysis. P<0.05 considered statistically significant. All the statistical analysis were carried out using the IBM SPSS Statistics for Windows, Version 20 (Released 2011; IBM Corp., Armonk, New York, United States).

## Results

The ANOVA revealed statistically significant differences in the study groups. These included the control, aspirin (ASP; 150 mg/kg), and aqueous (AFZ) and methanolic (MFZ) extracts of fresh rhizome of *Z. officinale *(100 mg/kg and 200 mg/kg each). Compared to the control group, Dunnett’s multiple comparison tests revealed a statistically significant difference in the time taken for the tail-flick response at 30, 60, and 120 mins for all the treatment groups (P<0.05) except for AFZ 100 mg/kg at 30 min. AFZ 100 mg/kg showed no statistically significant difference in the time taken for tail-flick response at 30 min compared to the control group (P>0.05). The Bonferroni Multiple Comparison tests revealed no statistically significant difference in the time taken for the tail-flick response with AFZ and MFZ compared to aspirin at 30, 60, and 120 min (P>0.05), except for AFZ 100 mg/kg dose at 30 min and 60 min (Table 2).

**Table 2:** Effect of ASP 150, AFZ 100, AFZ 200, MFZ 100, and MFZ 200 treatments on the rat tail-flick response compared to the control group (normal saline) All doses are in mg/kg; ASP: Aspirin; AFZ: Aqueous extract of fresh rhizome of *Zingiber officinale; *MFZ: Methanolic extract of fresh rhizome of *Zingiber officinale; *min: minute; P: Probability factor *post hoc analysis by Dunnett’s test ( p<0.05)

Time after treatment	Time of tail flick (seconds)						ANOVA
	Control	ASP 150	AFZ 100	AFZ 200	MFZ 100	MFZ 200	P Value
0 min	8.33 ± 0.816	8.33 ± 0.405	8.5 ± 0.547	8.66 ± 0.516	9.5 ± 0.836	9.16 ± 0.752	0.0218
30 min	10.33 ± 2.066	19.33 ± 0.816*	12.83 ± 1.835	14.5 ± 2.258*	17.5 ± 1.378*	17.33 ± 0.816*	<0.0001
60 min	11 ± 1.649	19.66 ± 0.516*	15.5 ± 2.429*	16.16 ± 3.212*	17.33 ± 1.633*	16.66 ± 0.816*	<0.0001
120 min	8.83 ± 1.722	16.33 ± 2.658*	13 ± 0.894*	13.83 ± 3.764*	16 ± 1.095*	15.16 ± 0.752*	<0.0001

## Discussion

In the present study, the analgesic effects of different doses of methanolic and aqueous extracts of fresh rhizome and dry powder of *Z. officinale* were tested in an experimental pain model. Extracts were prepared with the help of the Soxhlet apparatus. For the evaluation of analgesic activity, we selected the rat tail-flick method. The total number of rats used for this study was decided after reviewing the CPCSEA guidelines and previous studies to ensure the minimum number of animals needed to obtain statistically significant data.

Pain is a protective mechanism and tissue damage can lead to pain [2]. It is an unpleasant sensory and emotional experience associated with tissue damage. The currently used analgesic and anti-inflammatory agents like NSAIDS and corticosteroids, despite their high efficacy can lead to different adverse events generating major problems during their clinical use such as peptic ulcer, nephrotoxicity, hepatic damage, etc. [6]

There are a minimum of 115 constituents identified from fresh and dried ginger, among which gingerols are the major ones. They are abundant in fresh ginger and less so in dry ginger whereas shogaols are more abundant in the latter than the former. Shogaols are referred to as the major dehydration products of gingerols [13].

There are different possible mechanisms for the analgesic and anti-inflammatory effects of ginger. It has the capacity to inhibit prostaglandin and leukotriene biosynthesis [14]. It is suggested that the inhibition of arachidonate 5-lipoxygenase also helps in the anti-inflammatory activity of ginger [15]. Inhibition of cyclooxygenase 2 [16] and proinflammatory cytokines by gingerol [17] is also claimed to be responsible for its anti-inflammatory activity. Similar mechanisms may also be responsible for its analgesic effect. Considering these results, the evaluation of the analgesic activity of extracts of dry and fresh ginger is proposed.

AFZ 100 mg/kg and AFZ 200 mg/kg

We found that AFZ 100 mg/kg and 200 mg/kg (AFZ 100 and AFZ 200) showed statistically significant differences in the time taken for rat tail flick (p<0.05) when compared to the control at 30 min, 60 min, and 120 min. When we compared AFZ 100 and AFZ 200 with aspirin, the time taken for the rat tail-flick response (p<0.05) was statistically significant at all time intervals except for AFZ 200 at 120 min. Hence, we concluded that the analgesic activity of AFZ 200 is comparable with that of aspirin only at 120 min. When the AFZ was increased from 100 mg/kg to 200 mg/kg, we observed an increase in the time taken for the rat tail-flick response. So, we concluded that AFZ shows dose-dependent analgesic activity, though it is not as effective as aspirin.

MFZ 100 mg/kg and MFZ 200 mg/kg

We found that MFZ 100 mg/kg and 200 mg/kg (MFZ 100 and MFZ 200) showed statistically significant differences in the time taken for the rat tail-flick response (p<0.05) compared to the control at 30 min, 60 min and 120 min. When we compared MFZ 100 and MFZ 200 with aspirin, no statistically significant difference was seen in time taken for the rat tail-flick response (p<0.05) at the 30- and 120-minute intervals. Hence, in general, we concluded that their analgesic activity was comparable with aspirin. When MFZ was increased from 100 mg/kg to 200 mg/kg, there was no increase in the time taken for the rat tail-flick response. Hence, unlike AFZ, MFZ does not show dose-dependent analgesic activity.

Sepahvand et al. evaluated the analgesic activity of dry rhizome of ginger with the help of radiant heat-induced tail-flick test in rats [18]. They selected three different doses of ginger extracts (200 mg/kg, 400 mg/kg, and 600 mg/kg) and administered them intraperitoneally. They observed a decrease in nociception at all three doses of the extract. Their results showed that the duration of action increased with the increase in dose i.e., 200 mg/kg showed analgesic effect up to 60 minutes; 400 mg/kg showed analgesic effect up to 90 minutes, and 600 mg/kg showed analgesic activity up to 120 minutes. They also observed that the co-administration of ginger extracts with morphine produced significant analgesic effect compared to control and morphine alone. Our study showed comparable analgesic activity between ginger extract and control, but not comparable to aspirin. We also did not co-administer the extracts with aspirin. We suggest that the analgesic effect of aspirin may be potentiated by the ginger extract. Thus, we plan to study the analgesic and anti-inflammatory effects of the co-administration of ginger extract with aspirin or diclofenac in the future.

Another study by Raji et al. evaluated the analgesic activity of the rhizome extract of *Z. officinale* [19]. They selected acetic acid-induced writhing in mice as the test and administered ginger extract at doses of 50 mg/kg and 100 mg/kg intraperitoneally. Their results showed a decrease in the number of acetic acid-induced writhing at both doses compared to the control group. They also observed that the extract at the high dose of 100mg/kg was comparable to aspirin in its analgesic activity.

Ojewole et al. evaluated the analgesic activity of ethanolic extract of dried rhizome of *Z. officinale* [20] at 50, 100, 200, 400 and 800 mg/kg and administered them intraperitoneally. They selected two different models for evaluating analgesic activity in mice i.e. hot-plate and acetic acid test methods. They observed that* *the ethanol extracts showed significant analgesic effect in both the models when compared with the control and the standard drug group. Moreover, these extracts exhibited dose-dependent analgesic activity, which we also observed in case of AFZ in our study.

Thus, our results showed that the extracts of *Z. officinale* have analgesic property. In previous studies, we have shown that they exhibit anti-inflammatory activity too [21,22]. Considering the problems of gastric ulceration and hepatotoxicity associated with higher doses of aspirin and other NSAIDs, it will be justifiable to combine ginger extract with aspirin (or other NSAIDs) for chronic treatments of senile arthritis, low back ache, rheumatoid arthritis, etc. The analgesic activity of ginger might potentiate aspirin action, therefore we may be able to reduce dose of the latter. Moreover, due to its digestive and hepatoprotective properties, ginger might reduce the chances of aspirin-induced adverse effects. Therefore, we propose that clinical studies should be conducted to prove the benefit of co-administering ginger extract and NSAIDs in chronic pain conditions.

Limitations

The method and the parameters used to evaluate the analgesic activity were very primitive. Moreover, these activities were carried out in a single species i.e. Wistar rats. In our setup, we were unable to quantify the active principle present in the extracts. The efficacy of the extracts as analgesic agents can be further evaluated using other related models in different species and also in human studies.

## Conclusions

From the findings of the present experimental study, we conclude that the extracts of *Z. officinale* have analgesic activity. The 100 mg/kg and 200 mg/kg doses of AFZ and MFZ have significant analgesic activity compared to the control as assessed by the rat tail-flick model. Moreover, AFZ showed dose-dependent analgesic activity. The present study predicts that ginger may have beneficial analgesic effects in various pain conditions. This could be a step forward towards the formation of a new and safe drug that will be useful for patients suffering from pain, considering the adverse effects of conventional analgesic drugs such as NSAIDs and corticosteroids. Additionally, ginger extracts may be good adjuvant therapies along with aspirin or other NSAIDs due to their digestive and hepatoprotective activities. Further studies with ginger extracts need to be conducted on various analgesic models along with human studies to strengthen these results and prove their long-term efficacy as potential analgesic agents in routine clinical practice.

## References

[1] Robbins SL, Kumar V, Cotran RS (2020). Robbins & Cotran Pathologic Basis of Disease. Robbins and Cotran pathologic basis of disease.

[2] Becker DE (1996). Pain management in adult dental patients: the art and science of successful regimens. Pract Periodontics Aesthet Dent.

[3] Hall JE, Hall ME (2020). Guyton and Hall Textbook of Medical Physiology. https://shop.elsevier.com/books/guyton-and-hall-textbook-of-medical-physiology/hall/978-0-323-59712-8.

[4] Oxenham D (2023). Davidson’s Principles and Practice of Medicine. Davidson’s Principles and Practice of Medicine.

[5] Longo D, Fauci A, Kasper D, Hauser S, Jameson J, Loscalzo J (2011). Harrison's Principles of Internal Medicine, 18th Edition. Harrison's Principles of Internal Medicine.

[6] Elhwuegi AS, Hassan KM (2012). The analgesic effect of different antidepressants combined with aspirin on thermally induced pain in Albino mice. Libyan J Med.

[7] Shirooye P, Mokaberinejad R, Ara L, Hamzeloo-Moghadam M (2016). Volatile constituents of ginger oil prepared according to Iranian traditional medicine and conventional method: a comparative study. Afr J Tradit Complement Altern Med.

[8] Zadeh JB, Kor NM (2014). Physiological and pharmaceutical effects of ginger (Zingiber officinale Roscoe) as a valuable medicinal plant. Eur J Exp Biol.

[9] Zahmatkash M, Vafaeenasab MR (2011). Comparing analgesic effects of a topical herbal mixed medicine with salicylate in patients with knee osteoarthritis. Pak J Biol Sci.

[10] Koçak İ, Yücepur C, Gökler O (2018). Is ginger effective in reducing post-tonsillectomy morbidity? A prospective randomised clinical trial. Clin Exp Otorhinolaryngol.

[11] Kravchenko I, Eberle L, Nesterkina M, Kobernik A (2019). Anti-inflammatory and analgesic activity of ointment based on dense ginger extract (Zingiber officinale). J Herbmed Pharmacol.

[12] Medhi B, Prakash A (2010). Practical Manual of Experimental and Clinical Pharmacology. Practical Manual of Experimental and Clinical Pharmacology.

[13] Jolad SD, Lantz RC, Chen GJ, Bates RB, Timmermann BN (2005). Commercially processed dry ginger (Zingiber officinale): composition and effects on LPS-stimulated PGE2 production. Phytochemistry.

[14] Srivastava KC, Mustafa T (1992). Ginger (Zingiber officinale) in rheumatismand musculoskeletal disorders. Med Hypotheses.

[15] Kiuchi F, Iwakami S, Shibuya M, Hanaoka F, Sankawa U (1992). Inhibition of prostaglandin and leukotriene biosynthesis by gingerols and diarylheptanoids. Chem Pharm Bull (Tokyo).

[16] Tjendraputra E, Tran VH, Liu-Brennan D, Roufogalis BD, Duke CC (2001). Effect of ginger constituents and synthetic analogues on cyclooxygenase-2 enzyme in intact cells. Bioorg Chem.

[17] Tripathi S, Bruch D, Kittur DS (2008). Ginger extract inhibits LPS induced macrophage activation and function. BMC Complement Altern Med.

[18] Sepahvand R, Esmaeili-Mahani S, Arzi A, Rasoulian B, Abbasnejad M (2010). Ginger (Zingiber officinale Roscoe) elicits antinociceptive properties and potentiates morphine-induced analgesia in the rat radiant heat tail-flick test. J Med Food.

[19] Raji Y, Udoh US, Oluwadara OO, Akinsomisoye OS, Awobajo O (2002). Anti-flammatory and analgesic properties of the rhizome extract of Zingiber officinale. Afr J Biomed Res.

[20] Ojewole JA (2006). Analgesic, antiinflammatory and hypoglycaemic effects of ethanol extract of Zingiber officinale (Roscoe) rhizomes (Zingiberaceae) in mice and rats. Phytother Res.

[21] Pakale PV, Khanwelkar CC, Jadhav SA (2022). Study of anti-inflammatory activity of aqueous and methanolic extracts of fresh rhizome of Zingiber Officinale in Wistar rats. Int J Health Sci.

[22] Pakale PV, Khanwelkar CC, Jadhav SA (2022). Study of anti-inflammatory activity of aqueous and methanolic extracts of dry powder of Zingiber officinale (SUNTH) in Wistar rats. Int J Health Sci.

